# Radium-223 and bone metastatic disease: still more to learn

**DOI:** 10.1093/jncics/pkad083

**Published:** 2023-11-11

**Authors:** Oliver Sartor

**Affiliations:** Department of Medical Oncology and Radiology, Mayo Clinic, Rochester, MN, USA

In this issue of the Journal, the article by Parker and colleagues (1) addresses responses in bone metastatic lesions after administration of radium-223 to patients with castration-resistant prostate cancer. Given the difficulty of response assessment in osteoblastic bone lesions and the lack of clarity on how radium-223 might exert antineoplastic action after binding to lesions in the bone metastatic microenvironment, these data are of interest from several perspectives.

First, let us consider radium-223. This isotope was the first ɑ emitter to receive US Food and Drug Administration approval in any disease. It was also the first bone-targeted agent to prolong survival in any cancer (2). Radium-223 does not bind to cancer cells but rather binds the inorganic bony matrix found in abundance in osteoblastic stroma (3). Prostate cancer metastases, as is well known, is a bone-tropic disease, and in the typical patient osteoblastic lesions predominate. Though lytic bone lesions are occasionally encountered, particularly in various aggressive variants, prostate cancer bone metastases are typically osteoblastic and associated with calcium deposition on computed tomography scans and increased uptake of radiotracers when using bone scintigraphy (bone scans) with imaging agents such as Tc-99 methylene diphosphonate. Measuring response in osteoblastic bone lesions has traditionally been viewed as problematic. Using Response Evaluation Criteria in Solid Tumors criteria (4), such lesions are not evaluable for response. Rather than response, progression is captured by using serial bone scan assessments (5). Progression criteria use the development of new lesions rather than the enlargement of lesions previously detected.

Parker et al. (1) measured responses in bone metastatic lesions using whole-body diffusion–weighted magnetic resonance imaging (DWI). This technique has previously been used for assessing response (and progression) in both bone and other metastatic sites. With DWI, we can assess the mobility of water in tissue. Prior studies indicated that the mobility of water is diminished in cancerous tissues, likely due to cellular density. This mobility can be measured quantitatively by the apparent diffusion coefficient. When cellularity of tumors diminish, the apparent diffusion coefficient goes up. Such apparent diffusion coefficient measurements have previously been linked to tumor response. This technique was exploited by Parker and colleagues to assess response/progression in bone lesions following radium-223 treatments. No prior reports have used this technique in assessments of radium-223 efficacy.

Radium-223 prolongs overall survival in bone-dominant metastatic castration-resistant prostate cancer, as shown in the phase 3 ALSYMPCA trial (2). The trial assessed neither progression nor response in bone metastases. Prostate-specific antigen (PSA) measurements, a biochemical response assay, indicated low PSA response rates. Taken together, though the phase 3 radium trial was unequivocally positive for survival, PSA and image-based correlates were lacking. One alteration after radium-223 has been noted: Serum alkaline phosphatase typically goes down (6). In patients with prostate cancer and bone metastases, serum alkaline phosphatase is typically elevated because of the osteoblastic activity observed in osteoblastic metastatic disease. Interestingly, the interplay between the activated osteoblast in the bone stromal microenvironment and the cancerous metastatic deposit create a “vicious cycle,” with the cancer activating the surrounding bone stroma and activated bone stroma providing an environment conducive to enhancing cancerous growth (7).

So, what did Parker et al. (1) find in their novel exploration of whole-body DWI assessments following treatment with radium-223 in metastatic castration-resistant prostate cancer? First, they noted “marked heterogeneity of response, both between different patients and between different metastases within the same patient.” This finding conclusively establishes that not all the metastases are the same in radium-223-treated patients. Such heterogeneity is not unexpected given that genetic heterogeneity is well established in castration-resistant prostate cancer (8). The fact that sharp distinctions exist after treatment with an ɑ emitter is notable and important, however. Does this finding reflect variations in radium-223 uptake in individual lesions or variations in the tumor response to similar doses of ɑ radiation? Additional studies are required to understand those distinctions. Second, the authors noted that response, defined as a 30% increase in global median apparent diffusion coefficient, was seen in 14 of 36 (39%) patients. Clearly, antitumor responses were detected following treatment with radium-223 using the magnetic resonance imaging (MRI) technologies deployed. This finding represents an important and novel finding. For some patients, the response occurred after a single dose of radium, which begs the question: Which lesions regress after radium-223 and why? In addition to the global mean apparent diffusion coefficient measurements, up to 5 representative bone target lesions larger than 2 cm in size were selected for repetitive assessment. Based on a 30% increase in mean apparent diffusion coefficient of the 5 target lesions, response was seen in 23 of 36 (64%) patients. Does this finding imply that the larger lesions (presumably more established lesions) were more effectively treated by radium-223? Likely, that is so. Third, new bone metastatic lesions were noted during treatment in 26 of 36 (72%) patients. These new bone metastases were not present on the baseline MRI scans. Does this suggest that new metastatic lesions may not have sufficient bone stromal deposition of radium-233? Regardless of mechanism, this finding clearly calls for radium-223 to be used in combination with other effective therapies capable of controlling new lesion formation. What about correlations between biochemical measurements and MRI response? Interestingly, there was a clear correlate for PSA response and DWI improvement but no clear relationship between alkaline phosphatase response and DWI. For this observer, the relationship between PSA decline and DWI underscores the potential importance of the MRI findings observed herein. In terms of overall survival, there was no relationship between global mean apparent diffusion coefficient response and survival, but a qualitative assessment of response using METastasis Reporting and Data System for Prostate Cancer criteria (9) and overall survival demonstrated a statistically significant relationship. This finding is provocative, but clearly larger studies are required to confirm these relationships.

Taken together, the assessment of bone metastatic lesions by whole-body DWI is an important tool that investigators can employ when evaluating radium-223 and a variety of newer therapies. A methodology such as that employed by Parker and colleagues gives valuable insights into radium-223 actions and helps us understand the heterogeneity of the treatment effects. Given the evolutionary nature of cancers during treatment and the challenges that investigators face in developing new treatments, the addition of whole-body DWI assessments could potentially accelerate drug development by providing additional insights into the complex relationship between individual tumors and therapeutic response. More studies are clearly needed.

## Data Availability

No new data were generated or analyzed for this editorial.
